# New Bioelectrical Impedance-Based Equations to Estimate Resting Metabolic Rate in Young Athletes

**DOI:** 10.3390/mps8030053

**Published:** 2025-05-19

**Authors:** Theodoros Stampoulis, Alexandra Avloniti, Dimitrios Draganidis, Dimitrios Balampanos, Polyxeni Efthimia Chalastra, Anastasia Gkachtsou, Dimitrios Pantazis, Nikolaos-Orestis Retzepis, Maria Protopapa, Athanasios Poulios, Nikolaos Zaras, Maria Michalopoulou, Ioannis G. Fatouros, Athanasios Chatzinikolaou

**Affiliations:** 1 Department of Physical Education and Sport Sciences, School of Physical Education, Sport Sciences and Occupational Therapy, Democritus University of Thrace, 69100 Komotini, Greece; tstampou@phyed.duth.gr (T.S.); dimibala10@phyed.duth.gr (D.B.); polychal2@phyed.duth.gr (P.E.C.); anasgkac1@phyed.duth.gr (A.G.); dpantazi@phyed.duth.gr (D.P.); nretzepi@phyed.duth.gr (N.-O.R.); mprotopa@phyed.duth.gr (M.P.); nzaras@phyed.duth.gr (N.Z.); michal@phyed.duth.gr (M.M.); achatzin@phyed.duth.gr (A.C.); 2 Department of Physical Education and Sport Science, School of Physical Education, Sport Science and Dietetics, University of Thessaly, 42100 Trikala, Greece; ddraganidis@uth.gr (D.D.); athanpoul@gmail.com (A.P.); ifatouros@uth.gr (I.G.F.)

**Keywords:** body composition, trained population, bioelectrical impedance

## Abstract

Resting metabolic rate (RMR) significantly impacts total daily energy expenditure, particularly on training days, and varies among trained individuals. Studies estimating RMR in this population show notable discrepancies. This study aimed to develop and validate new bioelectrical impedance analysis-based (BIA) RMR equations for young athletes, using a calibration and a validation group of 219 and 51 participants, respectively. RMR was measured via indirect calorimetry, while body composition was assessed through DXA and BIA. Correlation and agreement were evaluated by using Pearson’s correlation coefficients and Bland–Altman analysis. Multiple linear regression was applied for the estimation of RMR and a one-way ANOVA was used to compare the new BIA-based equations with other specific formulas. A significant correlation was noted between the BIA and DXA measurements. The final equation, applicable to both genders, was significantly correlated with intracellular water (ICW) and trunk fat, predicting 71.1% of RMR variance. When analyzed separately, body weight and protein displayed a moderate correlation with RMR in men (r = 0.616, *p* < 0.001), while ICW was correlated with the percentage of body fat in women (r = 0.579, *p* < 0.001). In the validation group, the values obtained through the three BIA-based equations were similar to the measured RMR, but differed significantly from those obtained through the four existing equations for trained individuals. In conclusion, the developed equations based on BIA-mediated body composition analysis provide a reliable method for estimating RMR in trained populations daily.

## 1. Introduction

Total daily energy expenditure (TDEE) includes the resting metabolic rate (RMR), the thermic effect of physical activity and/or exercise, and diet-induced thermogenesis. RMR represents the amount of energy that is required to maintain vital functions at rest and accounts for 60–75% of TDEE in sedentary individuals [1,2,3,4] and up to 50% in athletes [4]. Factors such as age, gender, dietary intake, body composition, and physical activity level have been shown to affect RMR and result in this large variability [5,6].

Athletes participate systematically throughout the year in training sessions, including cardiorespiratory and resistance exercises that can alter their RMR over time [7,8]. In addition, it is evident that athletes display higher fat-free mass (FFM) than sedentary individuals [2]. Given the well-documented correlation between FFM and RMR, the higher FFM observed in athletes compared to sedentary individuals might explain the higher RMR observed in the former compared to the latter, in both genders [9].

To optimize training-induced adaptations on performance and body composition [9], athletes and those engaged in regular exercise need to manage their daily dietary intake according to their energy expenditure to achieve an energy balance, an energy surplus, or an energy deficit, depending on the training goal (i.e., maximize physical performance, reduce body weight, increase muscle mass, etc.) [10]. To this aim, the estimation or even prediction of RMR, as a crucial regulator of TDEE [11,12], is vital for this population.

Indirect calorimetry is the most widely used method for estimating RMR [13,14]. However, it requires specific conditions and specialized equipment to ensure accurate and reliable assessments, which may not always be feasible [15,16,17]. These limitations make its implementation challenging in field settings and particularly during daily routines. Therefore, various RMR prediction equations incorporating variables such as body weight, body height, age, body fat, and FFM have been developed and widely used to estimate RMR [5,18]. The most common equations, including those of Harris–Benedict [19], FAO/WHO/UNU [20], Cunningham [21], and Schofield [22], have been developed based on measurements in either sedentary individuals or individuals with low physical activity levels. As a consequence, these equations underestimate RMR in athletic populations [14,23] and display an agreement of less than 60% with the measured RMR [24,25].

In light of this discrepancy, an increasing number of new equations have been developed over the last 10 years based on athletic populations. However, most of them display considerable heterogeneity in terms of the sample size [26,27,28], participants’ physical conditioning level (participants with disparate physical condition levels ranging from elite or sub-elite athletes to individuals engaging in different sports/sport activities [13,23,27]), gender (a few studies included only males or females [23,29]) and race [30], making the validity and reliability of these equations questionable. According to a recent systematic review, both De Lorenzo’s and ten Haaf’s equations demonstrated acceptable agreement with measured RMR values, but they are missing a formal validation testing [24]. A recent meta-analysis suggested that ten Haaf’s equation may offer superior predictive potential in athletic populations, emphasizing the importance of selecting RMR prediction equations according to specific characteristics of the targeted group [25]. To our knowledge, only two studies that developed RMR prediction equations for individuals engaged in sport activities, have conducted a formal validation process to assess their accuracy and reliability [31,32]. However, none of them have examined the validation of BIA devices, to estimate body composition, against the gold standard method of DXA in the participants [24].

Undoubtedly, the development of an accurate and validated RMR prediction equation for athletes and trained males and females is required. Such a prediction equation will allow sports nutritionists and practitioners to estimate the energy needs of this population accurately and to prevent an energy imbalance that may not only hamper the training-induced adaptations and performance enhancement [10], but also affect metabolic health [33]. The aim of this study was to develop new RMR prediction equations for young, trained males and females based on the bioelectrical impedance analysis (BIA) of body composition and to validate them in a group of age-matched trained counterparts. The reason for selecting BIA to assess body composition is that this portable instrumentation provides an objective, cost-effective, and radiation-free methodological approach for estimating FFM in field-based environments. Furthermore, in contrast to skinfold measurement, BIA eliminates the operator-dependent variability and considering its strong correlation with DXA instrumentation in non-athlete populations [34,35,36,37], it does not necessitate the use of such types of high-cost imaging technologies.

## 2. Materials and Methods

### 2.1. Participants

According to a preliminary power analysis (power 0.95, an error probability 0.05, H1 *ρ*^2^ 0.3, and 18 predictors) that was performed using the G*Power Software (Version 3.1.9.4.), a minimum sample size of 89 participants was required to detect statistically meaningful differences in the linear multiple regression random model. Therefore, a total of 219 males (*N* = 104, age: 20.52 ± 1.57 years, weight: 79.74 ± 10.19 kg, height: 181.3 ± 7.25 cm, BMI: 24.24 ± 2.55 kg/m^2^, body fat mass: 12.17 ± 5.55 kg) and females (*N* = 115, age: 20.20 ± 1.35 years, weight: 60.67 ± 7.86 kg, height: 165.81 ± 7.86 cm, BMI: 22.05 ± 2.44 kg/m^2^, body fat mass: 15.92 ± 4.85 kg) who reported a training age of ≥5 years and a training frequency of ≥4 days per week for a minimum of 2 h per training session were included in the present study as the calibration group. Participants were engaged in different sports and were classified as highly trained [38]. Exclusion criteria included cardiac, respiratory, circulatory, metabolic, psychiatric, immune, autoimmune, endocrinological, hematological, neurological, or musculoskeletal disorders or diseases. A total sample of 51 age-matched, counterparts were recruited to be the validation group: males (*N* = 24, age: 21.04 ± 1.73 years, weight: 84.57 ± 11.38 kg, height: 181.75 ± 8.84 cm, BMI: 25.21 ± 3.19 kg/m^2^, body fat mass: 15.23 ± 7.80 kg) and females (*N* = 27, age: 20.11 ± 1.37 years, weight: 60.61 ± 8.61 kg, height: 165.93 ± 5.88 cm, BMI: 21.81 ± 2.41 kg/m^2^, body fat mass: 15.54 ± 4.63 kg). Data from these individuals were not used in the development of the equation; they were exclusively employed for its validation, thereby avoiding any potential bias. During their first visit, participants were fully informed about the aim and procedures of the study and provided their written informed consent. The study was approved by the Democritus University of Thrace Ethics Committee, and all procedures were aligned with the most recent version of the Declaration of Helsinki.

### 2.2. Study Design

Participants visited our lab facilities once to undergo an assessment of their anthropometrics (body weight and height), RMR and body composition using Dual Energy X-ray Absorptiometry (DXA), and BIA. The reason for using DXA to evaluate body composition was to improve the validity and quality of the study. BIA is widely used for assessing body composition among Caucasian athletes, but so far no prior studies have specifically evaluated the validity of BIA in trained individuals. Participants were previously instructed to abstain from any type of vigorous physical activity or exercise for ≥48 h prior to testing day to avoid the effect of excess post-exercise oxygen consumption (EPOC). In addition, females were instructed to visit the laboratory for the testing procedures between the 10th and 20th day of their menstrual cycle. On testing day, participants arrived at the laboratory early in the morning (7:30–10:00 a.m.) after an overnight fasting period of ≥8 h with an adequate level of hydration according to the bioelectrical impedance vector analysis (BIVA) and underwent the assessment with the following order: (i) anthropometrics, (ii) RMR, (iii) body composition using DXA, and (iv) BIA.

### 2.3. Anthropometrics

Participants’ body height (m) and weight (kg) were assessed using a wall-mounted stadiometer to the nearest 0.1 cm (SECA 206, SECA, Hamburg, Germany) and the bioelectrical impedance analysis device (CHARDER MA801, CHARDER, Taichung City, Taiwan) to the nearest 0.1 kg, respectively. Both measurements were performed with participants wearing their underclothes and barefoot. Body mass index (BMI) was calculated via the following equation: BMI = body weight (kg)/height (m)^2^.

### 2.4. Dual Energy X-Ray Absorptiometry (DXA)

In this study, the Lunar iDXA machine (GE Healthcare, Madison, WI, USA) was used to evaluate participants’ Whole Body Muscle Mass (WBMM) and Appendicular Skeletal Muscle Mass (ASMM). Before proceeding with the measurement, participants were asked to remove any metal objects to prevent any interference during the measurement. Participants lay on the scanning table in a supine position during the whole body DXA scan. Sensors placed across from the X-ray source detected the attenuation of low-dose X-ray beams passing through the body. This procedure facilitated the identification of fat-free mass (FFM) and body fat mass (FM).

### 2.5. Bioelectrical Impedance Analysis (BIA)

Body composition was assessed using (i) a five-frequency BIA system (CHARDER MA801, CHARDER, Taichung City, Taiwan). During the assessment with BIA, participants stood barefoot on the electrode plate and placed their fingers on both ends of the grips surrounding the electrodes. They lowered their arms straight down in front of the body so that the arms and grips did not touch the body and legs. In addition, they had to bend their knees and elbows and remain motionless throughout the measurement (approximately 60 s), as described by the manufacturer.

### 2.6. Resting Metabolic Rate

RMR was assessed through indirect calorimetry (FITMATE Pro, Cosmed, Rome, Italy). Prior to measurement, participants were rested in a supine position for 30 min in a private room of the laboratory under normal temperature, dim lights, and quiescence. The canopy hood of FITMATE Pro was worn by participants, and then they stayed awake for 20 min to complete the assessment. The first 5 min of each test were discarded, and the measurement continued until the steady state period when the RMR coefficient of variation (CV) was ≤10%. Calibration was performed before each measurement according to the manufacturer’s instructions [39]. To assess the test–retest reliability of the FITMATE Pro device, a subgroup of 27 participants (14 males, 13 females) from the calibration sample was evaluated on two separate occasions, five to six days apart, under the standardized conditions.

### 2.7. Estimation of Resting Metabolic Rate

Beyond indirect calorimetry, RMR was calculated using the two most appropriate equations for athletes: (i) De Lorenzo et al. [26] and (ii) ten Haaf and Weijs [27]. In addition, the equation of Jagim et al. [14] was included given the age-matched, multisport sample examined (Table 1).

### 2.8. Statistical Analysis

Demographic characteristics were presented as mean ± standard deviation (SD). The Kolmogorov–Smirnov and Shapiro–Wilk tests were used to assess the normality of data distribution. A stepwise linear regression analysis was conducted using BIA-derived variables (body fat mass, body fat percentage, skeletal muscle mass, segmental muscle and fat mass of the limbs, mineral mass, protein mass, intracellular and extracellular water), along with age, sex, and a constant, to develop the new BIA-based predictive equation for the total sample. Two additional regression models were developed separately for each gender using the same predictors. Variables were retained in the final models when the F-test *p*-value was below 0.05. One-way repeated measures ANOVA was employed to compare the measured RMR with the predicted RMR values derived from various equations across the total sample. Bland–Altman plots were used to assess the agreement and precision of the newly developed BIA-based equations (see Supplementary Materials). Paired-samples t-tests were performed to evaluate differences between the measured RMR and the predicted RMR values in the validation group. Additionally, correlation analyses were conducted between the BIA and DXA values for both fat mass and fat-free mass to assess measurement agreement, and Bland–Altman plots were generated to evaluate the level of agreement between these two methods. To assess the agreement between the measured (through indirect calorimetry) and estimated (through BIA-based equations) RMR, intraclass correlation coefficients (ICCs) were calculated using a two-way mixed-effects model for single measures with absolute agreement. To estimate the reliability of the Fitmate Pro RMR evaluation, a paired sample *t*-test and ICCs were calculated. Statistical significance was set at *p* ≤ 0.05. All data were analyzed using the Statistical Package for the Social Sciences (SPSS, Version 23.0; SPSS Inc., Chicago, IL, USA) [25].

## 3. Results

**Figure 1:**
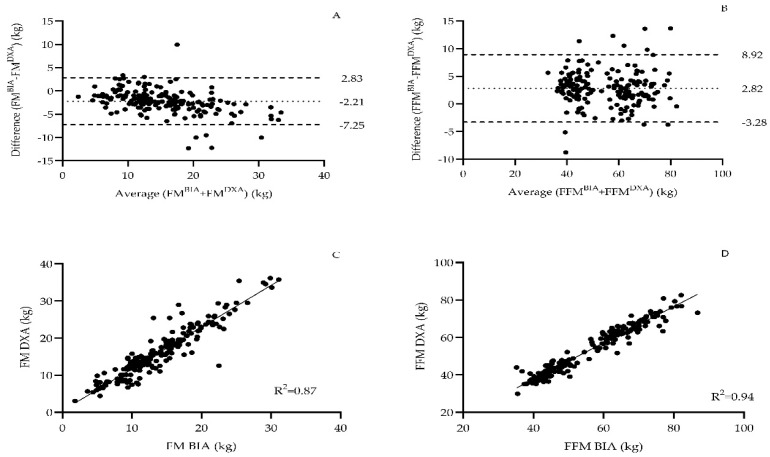
Bland-Altman plots were generated to illustrate the differences between dual-energy X-ray absorptiometry (DXA) and bioelectrical impedance analysis (BIA) measurements for body fat mass (FM) (**A**) and fat-free mass (FFM) (**B**), and correlation analyses were performed between the values (**C**,**D**) obtained from both methods in the total sample.

In the calibration group, a strong correlation was observed between the BIA- and DXA-based measurement of FM (r = 0.93, *p* < 0.05) and FFM (r = 0.97, *p* < 0.05). FM was underestimated by 2.21 ± 2.6 kg (*p* < 0.05), while FFM was overestimated by about 2.82 ± 3.1 kg (*p* < 0.05) in BIA compared to the DXA measurement (Figure 1).

Descriptive characteristics and BIA-derived data of the participants in the calibration group are shown in Table 2. Statistically meaningful differences were detected between males and females in anthropometrics and BIA-related parameters, except for age and trunk fat mass (TFM).

**Figure 2:**
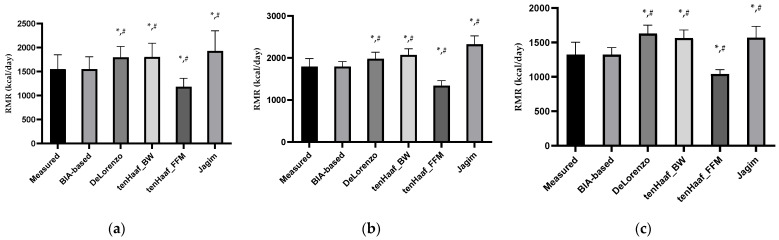
Comparison of measured and estimated resting metabolic rate in the calibration group (**a**), in men (**b**), and in women (**c**). * Differences between measured resting metabolic rate and the estimated RMRs, *p* < 0.05. # Differences between BIA-based estimated RMR and the other equations, *p* < 0.05.

Participants’ RMR, as assessed by indirect calorimetry and prediction equations, is presented in Table 3. Significant differences between males and females were noted (Figure 2).

### 3.1. Developing a New Bioelectrical Impedance Analysis (BIA)-Based Predictive Equation

**Figure 3:**
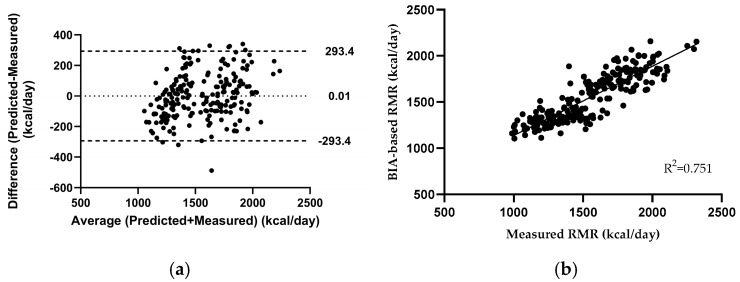
Bland-Altman plot for resting metabolic rate via BIA-based equation (**a**) and the relationship between measured and BIA-based predicted resting metabolic rate (**b**) for the calibration group.

The stepwise regression analysis showed that the best predictors with strong correlation (r = 0.867, *p* < 0.001) for RMR in the calibration group were intracellular water (ICW) and trunk fat mass (TFM) (R^2^ = 0.751, SEE: 150.39 kcal/day). Agreement between the two methods was assessed using the interclass correlation coefficient (ICC), based on a two-way mixed-effects model for single measures with absolute agreement. The resulting ICC was 0.92 (95% CI: 0.9–0.94) for the calibration group, and the proposed BIA-based equation was as follows (Figure 3):

RMR (kcal/day) = 394.509 + (42.869∗ICW) + (8.117∗TFM).

### 3.2. Development of New Gender-Dependent, Bioelectrical Impedance Analysis (BIA)-Based Equations

**Figure 4:**
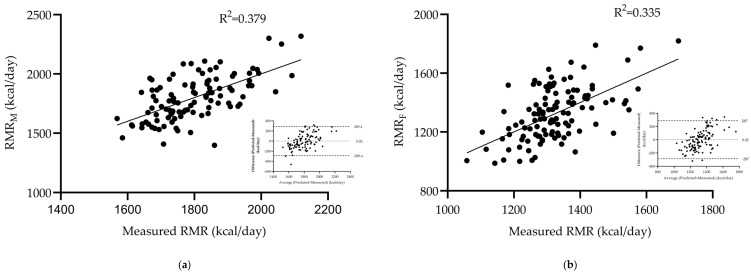
Relationship between measured and BIA-based gender specific predicted resting metabolic rate (RMR_M_ and RMR_F_) and Bland–Altman plots for resting metabolic rate via BIA-based equation for males (**a**) and for females (**b**) in the calibration group.

The stepwise regression analyses for each gender proposed that BW and protein (P) for males and ICW and body fat percentage (BFP) for females were the best predictors of RMR estimation. A strong correlation was retrieved for males (r = 0.616, R2: 0.379, SEE: 149.09 kcal/day) (Figure 4a) and a moderate one for females (r = 0.579, R2 = 0.335, SEE: 147.71 kcal/day) (Figure 4b), with the following equations presenting the relationships:

Males: RMRM (kcal/day) = 734.365 + [5.877∗BW (kg)] + [42.91∗P (kg)]

Females: RMRF (kcal/day) = 245.492 + [43.386∗ICW (L)] + [7.278∗BFP (%)]

The instrument demonstrated excellent test–retest reliability over the five to six days (ICC = 0.965) and Pearson’s r = 0.934 (*p* < 0.001), indicating strong temporal stability. The *t*-test results showed no systematic error among days (Day 1: RMR = 1816.3 ± 354.1 kcal vs. Day 2: RMR = 1773.48 ± 370.92, *p* = 0.105).

The validation group consisted of 51 athletes of both genders, and their characteristics are presented in Table 4. Statistically meaningful differences were detected between males and females in anthropometrics and BIA-related parameters, except for age, BFM, RHFM, LHFM, and TFM.

There were no significant differences between the measured RMR and the predicted RMR from the three BIA-derived equations (Table 5).

## 4. Discussion

The present study aimed at developing new BIA-based RMR prediction equations for trained individuals of both genders and to cross-validate them with an independent age- and gender-matched group of trained individuals. To our knowledge, this is the first study to develop RMR prediction equations that are independent of gender, body weight, and height, while including both hydration status and segmental fat mass, by using an instrumentation that is well-correlated with DXA. Most of the existing equations referring to both male and female athletes incorporate either FFM alone or body weight combined with body height, age, and gender for the estimation of RMR [27,39,40]. An exception is the equation developed by the muscular physique athletic population, which includes only body weight as a predictor parameter [28].

The predicted resting metabolic rate (pRMR) derived from the newly developed BIA-based equation demonstrated a strong correlation with the measured RMR (mRMR) (r = 0.867), with a mean energy deviation of approximately 150 kcal/day. This level of agreement aligns with findings by Marra et al. [23], who also employed BIA for body composition analysis in male athletes and reported a similar energy deviation of approximately 140–150 kcal/day across both of their predictive equations. When stratified by sex, the BIA-based equation developed in the present study showed a strong correlation in males (r = 0.616) and a moderate correlation in females (r = 0.579). These values are somewhat lower than those reported by ten Haaf and Weijs [27], who observed predictive accuracies of approximately 83–85% in males and 75–78% in females, despite reporting comparable energy deviations. In a related study, Jagim et al. [39] reported a predictive agreement of 53% with a standard error of estimate (SEE) of approximately 299 kcal/day for men, and a higher agreement of 77% with a SEE of 145 kcal/day for women. The larger variation observed in male compared to female athletes can be explained by the higher body mass and fat-free mass values of the male athletes, both of which are significant predictors of RMR [41]. Similarly, Tinsley et al. [28] reported strong correlations for both sexes with energy deviations around 170 kcal/day, which is consistent with the findings of the current study. The different methods utilized in body composition analysis could explain these disagreements. Actually, the first two studies used air displacement plethysmography [14,27], while the last one used DXA [29], which are laboratory methods and not practical in the field like BIA [13]. The selected parameters in this new BIA-based equation might be related to participants’ hydration status and body composition, as both genders displayed higher fat mass (males: 14.82% vs. 11.7%, females: 25.83% vs. 21.6%, respectively) compared to a similar multisport population [27]. Moreover, compared to a previous study by Jagim et al. [14] that included young athletes from different sports, in the present study, male participants demonstrated lower BMI (24.24 kg/m^2^ vs. 27.9 kg/m^2^), BFM (12.17 kg vs. 15.8 kg), and BW (79.74 kg vs. 93.7 kg) while females were characterized by higher BFM (15.92 kg vs. 14.1 kg). Another explanation could be the larger sample size in the present study as compared to previous reports, and the fact that it presented an almost equal number of both genders [23,27,28,40].

A secondary aim of the present study was to compare the developed equation with existing equations referring to the athletic population. De Lorenzo’s formula and ten Haaf’s weight-based equation overestimated the sample’s RMR while ten Haaf’s FFM-based equation underestimated it. Similarly, ten Haaf and Weijs reported that in a multisport young population of both genders, the equation developed by De Lorenzo underestimates mRMR similar to other equations that were based on non-athletic populations [27]. In young athletes from different sports, it was found that De Lorenzo’s equation overestimated RMR in females and underestimated it in males [14]. On the other hand, in a group of muscular physique athletes, both the weight-based equations of ten Haaf and De Lorenzo and the FFM-based formula of ten Haaf underestimated the athletes’ RMR [28]. Furthermore, in elite athletes, both De Lorenzo’s and ten Haaf’s BW-based equations overestimated the mRMR [13,23].

In the present study, BIA was validated against DXA within the same athletic population, demonstrating strong agreement across key body composition metrics, and thereby supporting the use of the BIA device as a reliable tool for assessing body composition, since the observed bias, due to underestimation or overestimation, falls within the range deemed acceptable by prior research [36,37]. Furthermore, in contrast to the vast majority of previous studies, including both genders, that had a total sample size of about one hundred athletes [27,39], the present study recruited a total sample size of 219 male and female participants Also, the present study utilized BIA to predict RMR, a practical field tool for daily use. The inclusion of the validation group reduced the risk of potential bias while ensuring a more impartial evaluation of the new BIA-based equations. Also, the present study reported the subjects’ training age, a parameter not reported in most previous studies. However, we should acknowledge as a limitation of the present study the fact that participants’ total training volume and frequency load characteristics and habitual physical activity level were not monitored, and the absence of multiple assessments at different time-points of the training seasons was neither recorded nor monitored.

## 5. Conclusions

In conclusion, the present study provides evidence that these new BIA-based RMR prediction equations might be a valid and accurate tool for estimating RMR in young athletes and trained individuals. As such, they can be efficiently utilized to enhance the daily control of energy demands for this population. However, to enhance the accuracy of the assessment, the BIA measurements should be conducted at least 48 h post-exercise, in order to minimize the influence of physical activity on body composition and RMR. In the future, research should focus on the development of advanced sport-specific RMR prediction equations for different age groups and on using more field-based methods of body composition analysis, like skinfolds or BIA.

## Figures and Tables

**Table 1:** Existing resting metabolic rate prediction equations.

Equations	Formula
De Lorenzo [27]	RMR (kcal/day) = −857 + [9∗BW (in kg)] + [11.7∗H (in cm)]
Ten Haaf_BW [28]	RMR (kcal/day) = 29.279 + ([11.936∗BW (in kg)] + [587.728∗H (in m)] − [8.129∗Age (years)] + [191.027∗Gender (M = 1, F = 0)])
Ten Haaf_FFM [28]	RMR (kcal/day) = 484.264 + [22.771∗FFM (in kg)]
Jagim_BW [38]	M: RMR (kcal/day) = 775.33 + [19.46∗BW (in kg)] F: RMR (kcal/day) = 288.6 + [21.10∗BW (in kg)]

BW: body weight; H: height; M: male; F: female; FFM: fat-free mass.

**Table 2:** Descriptive characteristics of the calibration group.

	Total (n = 219)		Male (n = 104)		Female (n = 115)		*p*-Value
	Mean	SD	Mean	SD	Mean	SD	
Age (years)	20.35	1.47	20.52	1.57	20.20	1.35	0.108
Height (cm)	173.16	10.17	181.3	7.25	165.81	5.94	<0.001
Weight (kg)	69.73	13.13	79.74	10.19	60.67	7.86	<0.001
BMI (kg/m^2^)	23.09	2.72	24.24	2.55	22.05	2.44	<0.001
BFM (kg)	14.14	5.51	12.17	5.55	15.92	4.85	<0.001
BFP (%)	20.60	7.55	14.82	5.27	25.83	5.09	<0.001
SMM (kg)	30.73	7.78	37.68	5.16	24.44	2.82	<0.001
Protein (kg)	11.60	5.63	13.85	1.40	9.56	7.07	<0.001
RHMM (kg)	3.05	0.97	3.93	0.58	2.26	0.40	<0.001
LHMM (kg)	3.01	0.96	3.88	0.58	2.23	0.38	<0.001
RHFM (kg)	0.59	0.30	0.47	0.28	0.70	0.25	<0.001
LHFM (kg)	0.58	0.29	0.46	0.28	0.69	0.25	<0.001
TMM (kg)	24.75	5.70	30.00	3.21	20.00	2.22	<0.001
TFM (kg)	6.95	3.01	6.75	3.19	7.14	2.84	0.334
RLMM (kg)	8.52	2.46	10.41	2.24	6.81	0.91	<0.001
LLMM (kg)	8.43	2.04	10.24	1.15	6.80	1.03	<0.001
RLFM (kg)	2.40	1.20	1.61	1.04	3.11	0.83	<0.001
LLFM (kg)	2.39	1.20	1.56	0.93	3.14	0.88	<0.001
ICW (L)	25.59	5.97	31.23	2.98	20.49	2.18	<0.001
ECW(L)	15.00	3.33	18.09	1.82	12.21	1.26	<0.001
PhA (degrees)	6.16	0.76	6.70	0.50	5.68	0.62	<0.001

BMI: body mass index; BFM: body fat mass; BFP: body fat percentage; SMM: skeletal muscle mass; RHMM: right-hand muscle mass; LHMM: left-hand muscle mass; RHFM: right-hand fat mass; LHFM: left-hand fat mass; TMM: trunk muscle mass; TFM: trunk fat mass; RLMM: right-leg muscle mass; LLMM: left-leg muscle mass; RLFM: right-leg fat mass; LLFM: left-leg fat mass, ICW: intra-cellular water; ECW: extra-cellular water; PhA: phase angle.

**Table 3:** Measured and estimated RMR through the equations in the calibration group.

	Total (n = 219)		Male (n = 104)		Female (n = 115)		*p*-Value
	Mean	SD	Mean	SD	Mean	SD	
**RMR (kcal)**							
Measured	1548.06	299.92	1797.45	187.34	1322.51	179.57	<0.001
BIA-based	1548.04	259.88	1797.45	115.32	1322.53	103.96	<0.001
De Lorenzo_BW	1796.55	224.68	1981.85	155.63	1628.97	122.47	<0.001
Ten Haaf_BW	1804.55	286.42	2070.83	148.94	1563.74	116.86	<0.001
Ten Haaf_FFM	1183.95	177.24	1342.16	117.52	1040.87	64.09	<0.001
Jagim_BW	1928.89	420.71	2327.08	198.29	1568.78	165.77	<0.001

RMR: resting metabolic rate; BIA: bioelectrical impedance analysis; BW: body weight; FFM: fat-free mass.

**Table 4:** Descriptive characteristics of the validation group.

	Total (n = 51)		Male (n = 24)		Female (n = 27)		*p*-Value
	Mean	SD	Mean	SD	Mean	SD	
Age (years)	20.55	1.60	21.04	1.73	20.11	1.37	0.037
Height (cm)	173.37	10.84	181.75	8.84	165.93	5.88	<0.001
Weight (kg)	71.88	15.62	84.57	11.38	60.61	8.61	<0.001
BMI (kg/m^2^)	23.41	3.26	25.21	3.19	21.81	2.41	<0.001
BFM (kg)	15.39	6.26	15.23	7.80	15.54	4.63	0.865
BFP (%)	21.65	7.33	17.38	6.84	25.44	5.50	<0.001
SMM (kg)	30.70	8.90	37.56	7.99	24.60	3.72	<0.001
Protein (kg)	11.41	2.93	14.21	1.11	8.93	1.35	<0.001
RHMM (kg)	3.11	1.01	4.06	0.44	2.27	0.50	<0.001
LHMM (kg)	3.04	0.99	3.95	0.47	2.23	0.49	<0.001
RHFM (kg)	0.65	0.33	0.63	0.41	0.66	0.24	0.684
LHFM (kg)	0.64	0.33	0.61	0.42	0.66	0.24	0.565
TMM (kg)	25.08	6.05	30.78	2.52	20.00	2.82	<0.001
TFM (kg)	7.71	3.58	8.51	4.39	7.00	2.56	0.135
RLMM (kg)	8.58	2.12	10.50	1.02	6.87	1.11	<0.001
LLMM (kg)	8.59	2.09	10.48	1.05	6.90	1.10	<0.001
RLFM (kg)	2.58	1.20	2.06	1.32	3.03	0.88	<0.001
LLFM (kg)	8.59	2.09	2.05	1.34	3.17	1.06	<0.001
ICW (L)	25.94	6.37	31.97	2.56	20.57	2.94	<0.001
ECW(L)	15.22	3.51	18.52	1.46	12.29	1.71	<0.001
PhA (degrees)	6.18	0.77	6.74	0.48	5.68	0.62	<0.001

BMI: body mass index; BFM: body fat mass; BFP: body fat percentage; SMM: skeletal muscle mass; RHMM: right-hand muscle mass; LHMM: left-hand muscle mass; RHFM: right-hand fat mass; LHFM: left-hand fat mass; TMM: trunk muscle mass; TFM: trunk fat mass; RLMM: right-leg muscle mass; LLMM: left-leg muscle mass; RLFM: right-leg fat mass; LLFM: left-leg fat mass; ICW: intra-cellular water; ECW: extra-cellular water; PhA: phase angle.

**Table 5:** Measured and estimated RMR through BIA-based equations in the validation group.

Total (n = 51)		Male (n = 24)		Female (n = 27)	
Measured	BIA-based	Measured	RMRM	Measured	RMRF
1621.84 ± 434.01	1568.91 ± 283.59	1943.00 ± 349.94	1841.03 ± 106.69	1336.37 ± 272.16	1323.12 ± 124.52
*p* = 0.165		*p* = 0.116		*p* = 0.767	

RMR: resting metabolic rate; BIA-based: bioelectrical impedance analysis based for total sample; RMRM: RMR equation for males; RMRF: RMR equation for females.

## Data Availability

The data used in this study, which includes information about participants, is confidential and cannot be shared due to stringent privacy regulations and ethical considerations. Access to the data is strictly restricted to the research team to protect the identity and well-being of the participants.

## Supplementary Materials

The following supporting information can be downloaded at: https://www.mdpi.com/article/10.3390/mps8030053/s1, Table S1: Bland-Altman Index.
