# Supplementary material for: New Bioelectrical Impedance-Based Equations to Estimate Resting Metabolic Rate in Young Athletes
Source: Methods Protoc. 2025 May 19;8(3):53. doi: 10.3390/mps8030053 (PMC12101231; doi:10.3390/mps8030053)
Supplement: Supplementary file 1 [file mps-08-00053-s001.zip › mps-3515953-supplementary.pdf]

Table S1. Bland Altman Index

	**Bias**	**95% LoA**
BIA-DXA FM	-2.21	-7.25- 2.83
BIA-DXA FFM	-2.82	-3.28-8.92
New-Based-FITMATE	0.01	-293.4 -293.4
RMRM-FITMATE	0.02	-289.4-289.4
RMRF-FITMATE	0.02	-287-287

BIA: Bioelectrical Impedance, DXA: Dual Energy X-Ray Absorptiometry, FM: Fat Mass, FFM: Fat-Free Mass, RMRM: Resting Meatbolic Rate Males, RMRF: Resting Metabolic Rate Females, LoA: Level of Agreement
